# Event Files are Common, But Semantic Event Metadata Remain Uneven in OpenNeuro BIDS Datasets

**DOI:** 10.1007/s12021-026-09797-y

**Published:** 2026-07-08

**Authors:** Yuxuan Xu

**Affiliations:** College of Computing, Georgia Institute of Technology, Atlanta, GA USA

**Keywords:** OpenNeuro, Brain imaging data structure, Event annotation, Hierarchical event descriptor, BIDS validator, Neuroinformatics

## Abstract

Open neuroscience repositories support reuse by making datasets accessible, but event level reuse also depends on whether task and stimulus annotations can be interpreted by software. I audited public latest OpenNeuro BIDS snapshots using public GraphQL metadata, recursive file trees, small events.json sidecars, and bounded events.tsv header ranges. Raw neural data were left untouched. Among 1,713 public latest snapshots, 1,483 had task metadata or observed event TSV files and formed the primary event relevant denominator. Event TSV files were present in 1,175/1,483 snapshots (79.2%; Wilson 95% CI 77.1%-81.2%). Candidate event JSON sidecars were present in 604/1,483 snapshots (40.7%), but an inheritance aware path and entity check found applicable JSON sidecars for 590/1,483 (39.8%), descriptive applicable sidecars for 550/1,483 (37.1%), and experiment specific applicable sidecars for 491/1,483 (33.1%). Across the full recursive file tree, 162,034/301,681 event TSV files (53.7%) had an applicable event JSON sidecar, and 147,412/301,681 (48.9%) had an applicable experiment specific sidecar. HED was detected in event JSON for 45/1,483 snapshots (3.0%) and in sampled TSV headers for 2/1,483 (0.13%). EEG had higher sidecar coverage and HED detection than fMRI, but 21.2% of EEG candidate JSON files were bookkeeping only. These results identify a repository visible metadata gap: event timing is common, but software interpretable event meaning remains uneven.

**Supplementary Information:**

The online version contains supplementary material available at https://doi.org/10.1007/s12021-026-09797-y.

## Introduction

The Brain Imaging Data Structure (BIDS) standardized how neuroimaging and related electrophysiology datasets are organized and described (Gorgolewski et al., 2016; Niso et al., 2018; Pernet et al., 2019; Holdgraf et al., 2019). OpenNeuro made BIDS public sharing operational at repository scale (Markiewicz et al., 2021), and NEMAR extended related infrastructure for neuroelectromagnetic data and compute workflows (Delorme et al., 2022). These resources are central to FAIR neuroscience because they improve dataset findability, access, and reuse infrastructure (Wilkinson et al., 2016). Still, repository level discovery by itself leaves open what each task event means.

The distinction matters for computational neuroscience. Many encoding models, decoding analyses, multimodal benchmarks, and automated meta-analyses require or benefit from reliable links between neural measurements and experimental events. BIDS event files describe timing and other event properties recorded during acquisition, and BIDS allows event JSON sidecars to describe event columns, categorical levels, and HED annotations (BIDS Specification, n.d.-a; BIDS Specification, n.d.-b). HED provides a formal vocabulary for software interpretable event annotation (Robbins et al., 2021, 2022). When such metadata are sparse, users often reconstruct task meaning from papers, README files, column names, or lab specific code.

This study audits a narrower question than dataset scientific quality or BIDS validity: when a public OpenNeuro latest snapshot appears event relevant, how often can a program find event timing files and an applicable event dictionary that describes event meaning? The revision explicitly addresses two possible counting errors. First, BIDS metadata may live at different levels of the directory tree, so the file tree must be walked recursively and interpreted with the BIDS inheritance principle. Second, a file named like an event sidecar may be empty, describe only generic bookkeeping columns, or contain unrelated provenance like content. The analysis therefore separates event TSV presence, candidate JSON presence, applicability to event TSV files, descriptive content, experiment specific content, and detectable HED.

## Results

### Corpus and Recursive File Audit

The OpenNeuro dataset connection returned 1,719 dataset IDs. Six records lacked a usable public latest snapshot or returned unrecoverable detail errors, leaving 1,713 public latest snapshots. The primary denominator was intentionally restricted to 1,483 event relevant snapshots with OpenNeuro task metadata or an observed event TSV file. A broader sensitivity denominator that also included fMRI, EEG, MEG, or iEEG datasets based on file family contained 1,523 snapshots and gave the same qualitative pattern. Recursive file tree retrieval failed for 2 public snapshots in the final regenerated run because the OpenNeuro GraphQL request returned a transient server error. Excluding those 2 snapshots changed the primary event annotation rates by about 0.1% point or less.

The primary event relevant corpus contained 922 fMRI, 410 EEG, 69 iEEG, 59 MEG, and 23 other task like snapshots. The recursive file tree audit detected 301,681 event TSV files and 55,914 candidate event JSON files in the primary denominator. Of these candidate JSON files, 55,912 parsed successfully; all datasets remained below the event JSON cap. Only 40 candidate JSON files had no event TSV target under the approximate inheritance check.

## Event Timing Exceeds Applicable Event Dictionaries

**Fig. 1:**
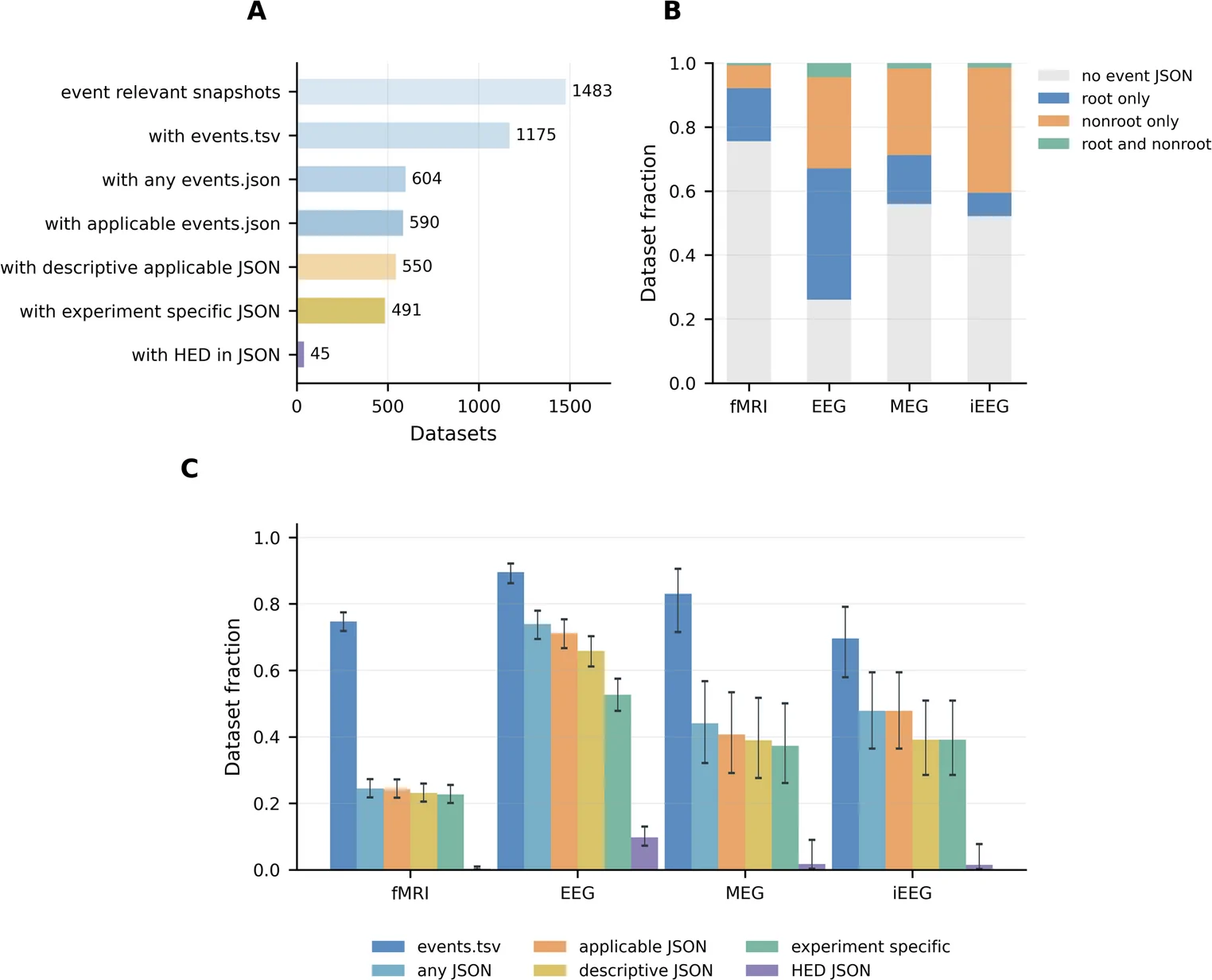
Recursive event metadata audit across OpenNeuro modalities. Denominator: 1,483 primary event relevant public latest snapshots with OpenNeuro task metadata or observed event TSV files. **A**: Dataset level cascade from event relevant snapshots to event timing, candidate event JSON, applicable event JSON, descriptive applicable JSON, experiment specific applicable JSON, and HED in JSON. **B**: Dataset level event JSON location after recursive scanning, grouped by modality and using exhaustive root and nonroot categories. **C**: Modality specific dataset fractions with Wilson 95% confidence intervals for event TSV presence, candidate event JSON presence, applicable JSON, descriptive applicable JSON, experiment specific applicable JSON, and HED in JSON

At the dataset level, event TSV files were present in 1,175/1,483 event relevant snapshots (79.2%, 95% CI 77.1%−81.2%). Candidate event JSON sidecars were present in 604/1,483 snapshots (40.7%, 38.3%−43.2%). After applying a BIDS style path/entity applicability check, 590/1,483 snapshots (39.8%, 37.3%−42.3%) had at least one event JSON sidecar applicable to an event TSV file. Descriptive applicable sidecars were present in 550/1,483 snapshots (37.1%, 34.7%−39.6%), and experiment specific applicable sidecars were present in 491/1,483 snapshots (33.1%, 30.8%−35.5%). Complete experiment specific coverage of all detected event TSV files within a snapshot was lower, at 460/1,483 snapshots (31.0%, 28.7%−33.4%) (Fig. 1A).

The file level results show why sidecar presence alone is too broad. Of 301,681 recursive event TSV files, 162,034 (53.7%) had an applicable event JSON sidecar, 157,885 (52.3%) had an applicable descriptive sidecar, and 147,412 (48.9%) had an applicable experiment specific sidecar. Thus, roughly half of detected event TSV files had a path applicable experiment specific candidate dictionary under this audit. These file level counts use filename/path applicability and sidecar content classification rather than row by row or column by column semantic verification for every TSV. They indicate that automated event level reuse would need additional human readable context or dataset specific interpretation for many files.

## Modality Differences Reflect Different Failure Modes

**Fig. 2:**
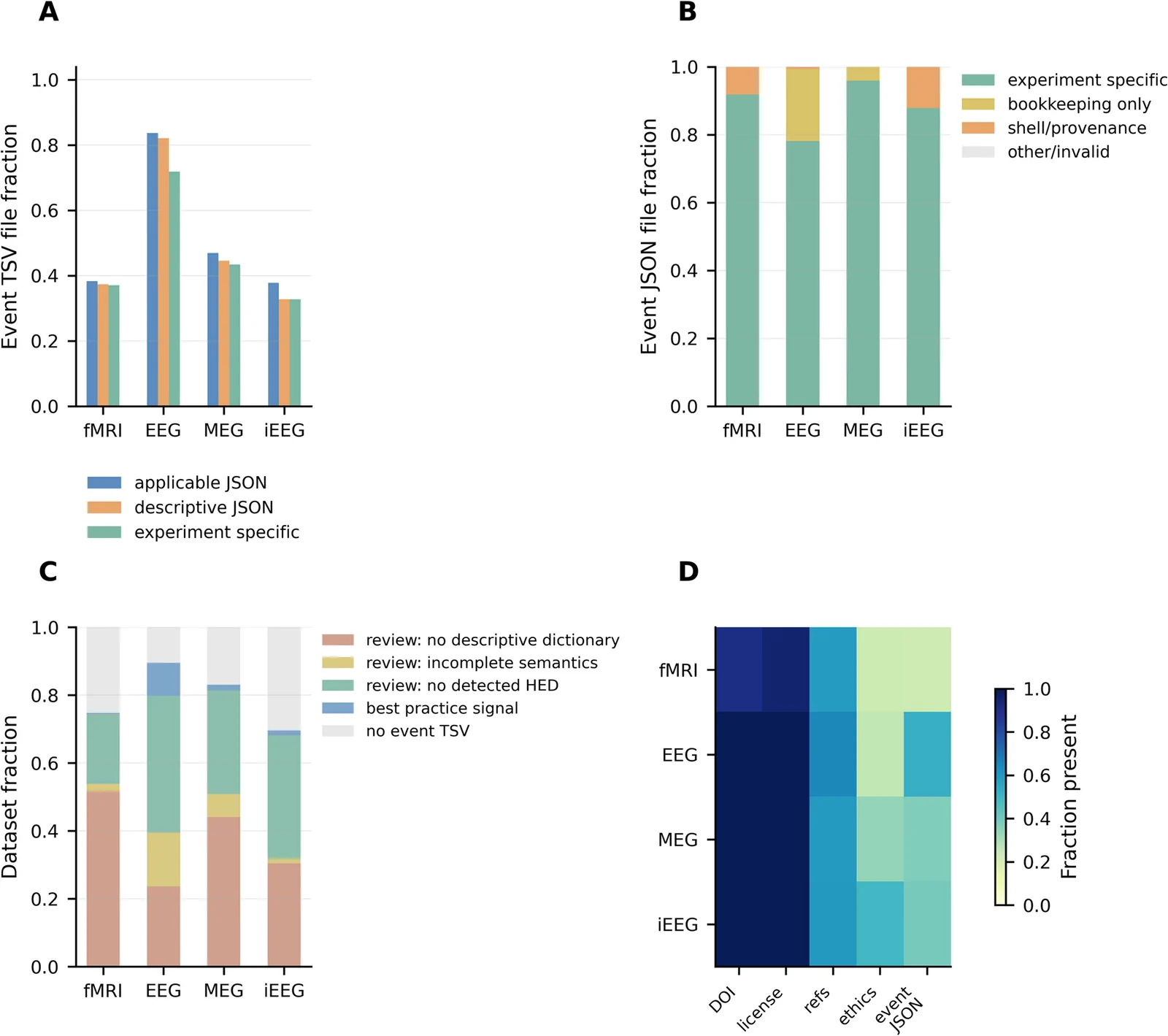
Recursive sidecar coverage, sidecar content, advisory prompts, and discovery metadata. **A**: Full recursive file level fraction of event TSV files with applicable JSON, descriptive applicable JSON, and experiment specific applicable JSON. **B**: Content classification of candidate event JSON files by modality. **C**: Prototype advisory categories intended as review prompts rather than binary pass or fail decisions. **D**: Dataset discovery metadata are more frequently detectable than experiment specific event JSON, especially for platform level DOI and license fields

The fMRI and EEG patterns differed. Event TSVs were detected in 689/922 fMRI snapshots (74.7%) and 367/410 EEG snapshots (89.5%). Candidate event JSON sidecars were detected in 225/922 fMRI snapshots (24.4%) and 303/410 EEG snapshots (73.9%). Experiment specific applicable sidecars were detected in 209/922 fMRI snapshots (22.7%) and 216/410 EEG snapshots (52.7%). File level coverage showed the same contrast: 37.1% of fMRI event TSV files and 71.9% of EEG event TSV files had applicable experiment specific sidecars (Fig. 2A). Table 1 summarizes these major modality dataset level and file level differences.

**Table 1:** Major modality recursive event sidecar audit

Modality	Snapshots	Events TSV	Any event JSON	Descriptive applicable JSON	Experiment specific applicable JSON	HED JSON	Event TSV files with experiment specific JSON	Bookkeeping only JSON files
fMRI	922	74.7%	24.4%	23.1%	22.7%	0.3%	37.1%	0.1%
EEG	410	89.5%	73.9%	65.9%	52.7%	9.8%	71.9%	21.2%
MEG	59	83.1%	44.1%	39.0%	37.3%	1.7%	43.4%	4.0%
iEEG	69	69.6%	47.8%	39.1%	39.1%	1.4%	32.8%	0.0%

Dataset level columns use the primary event relevant denominator within each modality. The file level column reports the fraction of all recursive event TSV files in that modality with an applicable experiment specific JSON sidecar. Bookkeeping only JSON files are fractions of candidate event JSON files in that modality. The primary denominator also included 23 other task like or mixed family snapshots; these are included in all overall counts but omitted from the table because they do not form a coherent acquisition modality.

The content of candidate JSON files also differed. Across candidate event JSON files in the primary denominator, 47,237/55,914 (84.5%) were classified as experiment specific dictionaries, 6,643/55,914 (11.9%) as bookkeeping only dictionaries, and 2,034/55,914 (3.6%) as shell or provenance like, invalid, or otherwise non descriptive files. Two candidate JSON files failed parsing and are included in the non descriptive count rather than in the descriptive or experiment specific counts. The bookkeeping only category was concentrated in EEG: 21.2% of EEG candidate event JSON files were bookkeeping only, compared with 0.08% in fMRI, 4.0% in MEG, and 0% in iEEG (Fig. 2B). This supports the reviewer’s concern that high sidecar frequency can still leave gaps in experiment specific semantic content.

Tool attribution could not be separated cleanly from these metadata alone. Event JSON sidecars lacked a reliable GeneratedBy field in this audit, and dataset level GeneratedBy fields, when present, could not identify which software wrote a particular event sidecar or which columns were manually curated afterward. README or file pattern proxies would also mix conversion, preprocessing, and repository packaging tools. The analysis therefore reports measurable content patterns while avoiding causal claims about particular communities or software packages.

## HED is Detectable in a Small Minority of Snapshots

HED was detected in event JSON for 45/1,483 event relevant snapshots (3.0%, 2.3%−4.0%). EEG accounted for most detected HED JSON cases: 40/410 EEG snapshots (9.8%). fMRI had 3/922 snapshots (0.33%), MEG had 1/59 (1.7%), and iEEG had 1/69 (1.4%). A literal HED column in sampled event TSV headers was detected in 2/1,483 snapshots (0.13%, 0.04%−0.49%). Because TSV headers were capped at 12 per dataset, HED column counts are bounded detectability estimates. By contrast, all small candidate event JSON sidecars were fetched for inspection in the final run, with two parse failures handled as non descriptive candidate files, so JSON based HED detection is the stronger estimate.

## Discovery Metadata and Event Semantics Measure Different Things

Dataset level discovery fields were more frequently detectable than event level semantic fields. In the primary event relevant corpus, 1,399/1,483 snapshots (94.3%) had a dataset DOI and 1,438/1,483 (97.0%) had a license. References were present in 896/1,483 (60.4%) and ethics approvals in 388/1,483 (26.2%). This comparison is best interpreted cautiously because it is not a direct measure of submitter effort. In OpenNeuro, versioned datasets receive persistent identifiers through the platform infrastructure, so DOI presence is partly a repository level feature rather than an event annotation feature. Validation warning counts were weakly associated with the descriptive event readiness score (Spearman rho = −0.067, *p* = 0.010), suggesting that syntactic validation and event semantic completeness capture different aspects of repository metadata.

### Prototype Advisory Categories

The reproducibility package includes a prototype advisory categorization intended as a guide rather than a binary pass or fail gate. In the primary denominator, 625 snapshots had event timing but no descriptive applicable dictionary, 90 had incomplete experiment specific coverage, 415 had complete experiment specific coverage without detected HED, and 45 had both complete experiment specific coverage and detected HED. The remaining 308 snapshots had no detected event TSV under the primary denominator. These categories are deliberately conservative prompts for human review. Their purpose is to encourage clear, source grounded event descriptions while discouraging inferred or superficial metadata that merely satisfies a checker. The dataset metrics table stores the advisory category for each snapshot, and the summary JSON stores aggregate counts.

## Discussion

This audit supports a constrained conclusion: OpenNeuro event timing files are common, but repository visible, software interpretable event semantics are less consistently present. The revised analysis walked the recursive file tree, checked whether candidate JSON sidecars could apply to event TSV files under BIDS style path/entity rules, and classified sidecar contents. The main result is that applicable, descriptive, experiment specific event dictionaries are present for about one third of event relevant snapshots and about half of recursive event TSV files.

The modality results suggest different practical bottlenecks. fMRI snapshots more often lacked event JSON sidecars, whereas EEG snapshots more often had sidecars but included a larger bookkeeping only fraction. HED was also much more visible in EEG than fMRI. One plausible explanation is workflow specific rather than biological: task fMRI event TSV files are often exported from experiment scripts and then attached to imaging data, while EEG conversion pipelines may emit event tables and sidecars from acquisition or analysis software defaults. Community infrastructure also differs; HED and NEMAR linked workflows have been especially visible in neuroelectromagnetic data sharing. These explanations are hypotheses about format and tool ecosystems rather than causal conclusions from the present audit. Tool and community effects require provenance information that is sparse in repository visible event sidecars.

The solution should be advisory. A useful validator or linter should report concrete review prompts instead of simply classifying datasets as good or bad: whether event TSV files exist, whether applicable event JSON sidecars exist under inheritance rules, whether sidecars parse, whether they describe event columns and levels, whether descriptions are experiment specific rather than bookkeeping only, and whether HED is present where appropriate. It could also surface ambiguous sidecars for human review or language model assisted triage, while leaving final interpretation to a human curator. Incorrect or invented metadata would be worse than clearly missing metadata, so the goal should be transparent guidance rather than superficial compliance.

The study also clarifies the role of OpenNeuro discovery metadata. DOI, license, and related fields make datasets more findable and citable, but they cannot guarantee event level semantic reuse. A dataset can be valid, citable, and scientifically valuable while still requiring manual work before automated event level analyses can be transferred across tasks, laboratories, or modalities.

## Methods

### Data Source

All data were obtained from public OpenNeuro metadata and object text endpoints. Dataset IDs and latest snapshot metadata were queried through the OpenNeuro GraphQL endpoint at https://openneuro.org/crn/graphql (OpenNeuro Documentation, n.d.-a). The file tree query explicitly requested files(recursive: true), returning file object IDs, names, sizes, and directory flags. The original index and dataset detail cache targeted public latest snapshots visible through the API on 2026-05-04 UTC. Revised outputs were regenerated on 2026-06-26 from the same cached dataset detail records, cached file trees where available, and public object text URLs.

The script downloaded only public metadata, recursive file records, small event JSON sidecars, and bounded event TSV text ranges for header inspection. It left raw neuroimaging, EEG, MEG, iEEG, NWB, MATLAB, and other raw neural data files untouched.

### Denominators

The primary event relevant denominator required OpenNeuro task metadata or an observed event TSV file. This reduces the chance of counting resting state or taskless functional datasets as lacking event annotations when event annotations may not be expected. A broader sensitivity denominator additionally included fMRI, EEG, MEG, or iEEG datasets based on modality or file family evidence. Both denominators are stored in results/openneuro_event_annotation_summary.json.

### Recursive Sidecar Matching

The script detected both exact events.tsv/events.json basenames and BIDS style _events.tsv/_events.json suffixes. Candidate event JSON sidecars were considered applicable to an event TSV file when the JSON file was stored at the same directory level or an ancestor level, shared the events suffix, and did not contain BIDS filename entities absent from or conflicting with the TSV filename. This is an approximate implementation of the BIDS inheritance principle. It is path/entity based and does not call a full BIDS parser.

### Event JSON Content Classification

Event JSON files smaller than 2 MB were fetched and parsed. All datasets remained below the final cap of 10,000 event JSON sidecars. A sidecar was classified as descriptive when it contained non empty column metadata such as Description, LongName, Levels, Units, TermURL, or HED. A string valued top level key was treated as weak legacy description evidence only when the key itself matched a recognizable semantic event column name; arbitrary string fields were insufficient. A descriptive sidecar was classified as experiment specific when at least one described column went beyond generic bookkeeping columns such as onset, duration, sample, latency, type, value, trial, run, block, or channel. Sidecars that described only such generic columns were classified as bookkeeping only. Sidecars without descriptive column evidence, including parse failures and non object JSON files, were classified as shell/provenance like or non descriptive candidate files. These labels are screening categories rather than judgments of scientific validity.

HED in JSON was detected by exact keys including HED, HEDVersion, HEDDefinitions, or HEDSchemaVersion. The detector intentionally avoided substring matches. A literal HED TSV column required a sampled header column exactly equal to HED after case normalization.

### TSV Header Sampling

TSV inspection used the first 8 KB of up to 12 representative event TSV files per dataset, ranked toward shallow/inherited paths and smaller files. These bounded text ranges may include early event rows, but the analysis used only the first line to evaluate header detectability. The TSV cap was exceeded by 1,056 datasets, and 8 datasets had sampled header fetch failures after sampling. Therefore TSV header HED and column overlap metrics are detectability estimates rather than exhaustive TSV content counts. Full TSV to sidecar applicability rates are computed from recursive filenames and JSON content classifications rather than the TSV header sample.

### Scores and Statistics

Primary conclusions use individual rates and recursive file level coverage rather than the composite score. The descriptive event readiness score is retained only as a screening summary across event TSV presence, applicable event JSON presence, task level coverage where task labels are available, descriptive JSON content, experiment specific JSON content, HED in JSON, HED TSV header detection, semantic TSV header detection, and observed onset/duration headers. Discovery completeness is a descriptive score across dataset DOI, license, authors, funding, references, dataset type, ethics approvals, BIDS version, participants.tsv, participants.json, and README presence; a secondary version excludes DOI to separate platform level persistent identifiers from other submitted metadata. Wilson 95% confidence intervals are reported for proportions. Spearman correlation compares validation warning counts with the descriptive event readiness score.

### Limitations

This audit measures repository visible metadata rather than the scientific quality of individual datasets or the truth of any event annotation. The inheritance check is an approximation based on paths and filename entities rather than a complete BIDS parser. TSV header sampling can miss rare HED columns or columns present only in unsampled runs. The content classifier is conservative but imperfect: a generic column name may still be meaningful in a specific dataset, and an experiment specific column description may still be too vague for a particular analysis. The audit leaves papers, README prose, source code archives, raw event values, simulations, and preprocessing tools outside its scope. It also cannot reliably separate human effort from tool generated metadata because event JSON sidecars lacked a stable tool field. Finally, results apply to OpenNeuro public latest snapshots in this audit rather than to private BIDS archives, non BIDS repositories, or all neuroscience data sharing.

## Supplementary Information

Below is the link to the electronic supplementary material.

Supplementary Material 1 Reproducibility package containing analysis scripts, requirements, derived CSV/JSON outputs, figures, manifest hashes, validation notes, and the manuscript source Markdown used to reproduce the reported audit tables and figures (ZIP 898 KB)

## Data Availability

Source dataset and latest snapshot metadata are publicly accessible through OpenNeuro and the OpenNeuro GraphQL API at https://openneuro.org/crn/graphql. The submitted Online Resource 1 supplementary package contains derived dataset level metrics, family summaries, summary JSON, figure outputs, code, requirements, validation outputs, and reproducibility notes. No raw neural data are redistributed.

## Code Availability

Online Resource 1 includes the analysis and validation code needed to reproduce the reported audit tables and figures. The validation checks required files, result table consistency, figure non blankness and PDF validity, manifest hashes when present, family summary consistency, and key audit invariants. The advisory prototype is implemented as analysis output rather than as a standalone BIDS Validator plugin; the dataset metrics table includes the advisory category for each snapshot.
