# Supplementary figures and images for: Event Files are Common, But Semantic Event Metadata Remain Uneven in OpenNeuro BIDS Datasets
Source: Neuroinformatics. 2026 Jul 8;24(3):40. doi: 10.1007/s12021-026-09797-y (PMC13346197; doi:10.1007/s12021-026-09797-y)

**A**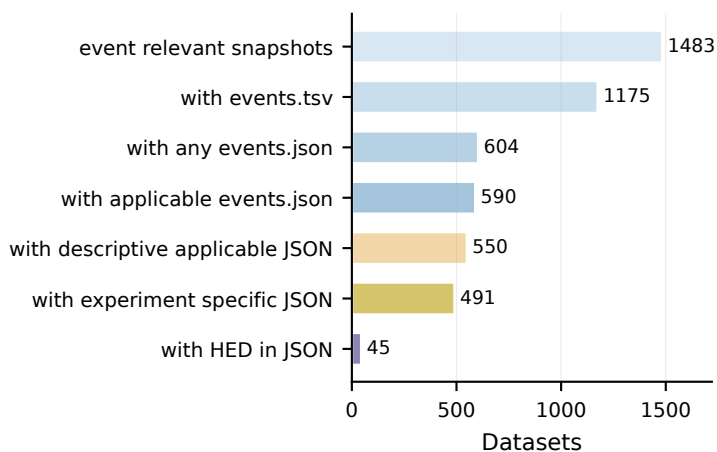**B**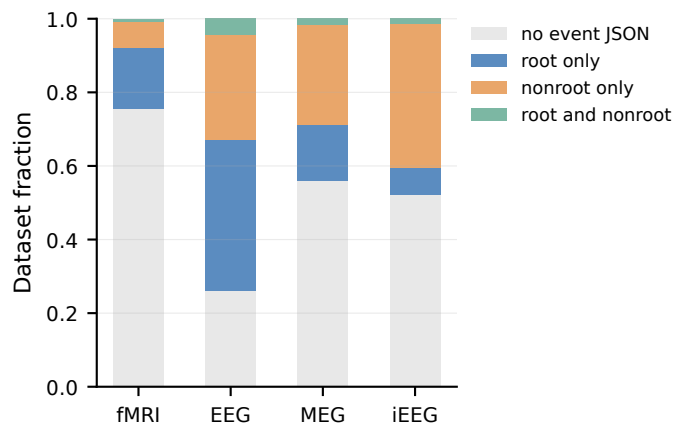**C**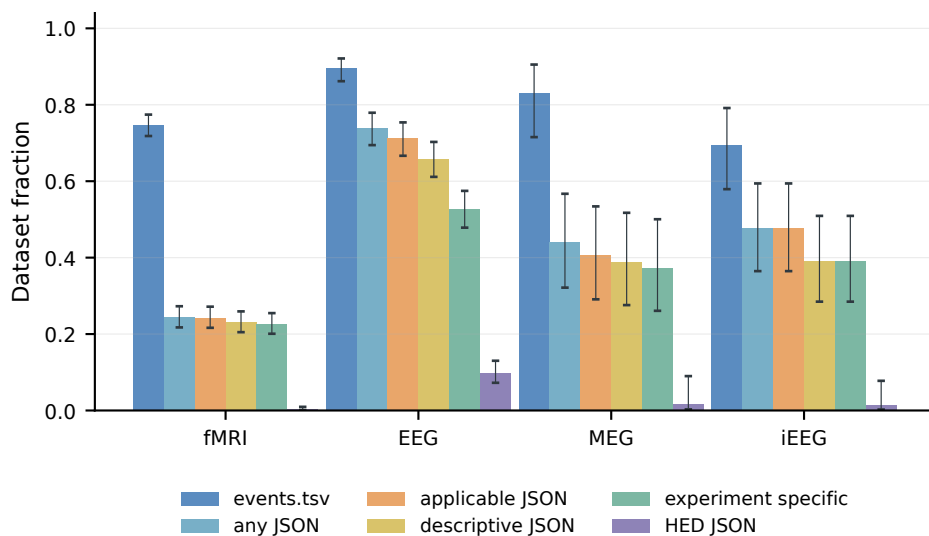

Supplement: Supplementary file 1 — Supplementary Material 1 Reproducibility package containing analysis scripts, requirements, derived CSV/JSON outputs, figures, manifest hashes, validation notes, and the manuscript source Markdown used to reproduce the reported audit tables and figures (ZIP 898 KB) [file 12021_2026_9797_MOESM1_ESM.zip › results/figures/figure1_event_readiness.pdf]

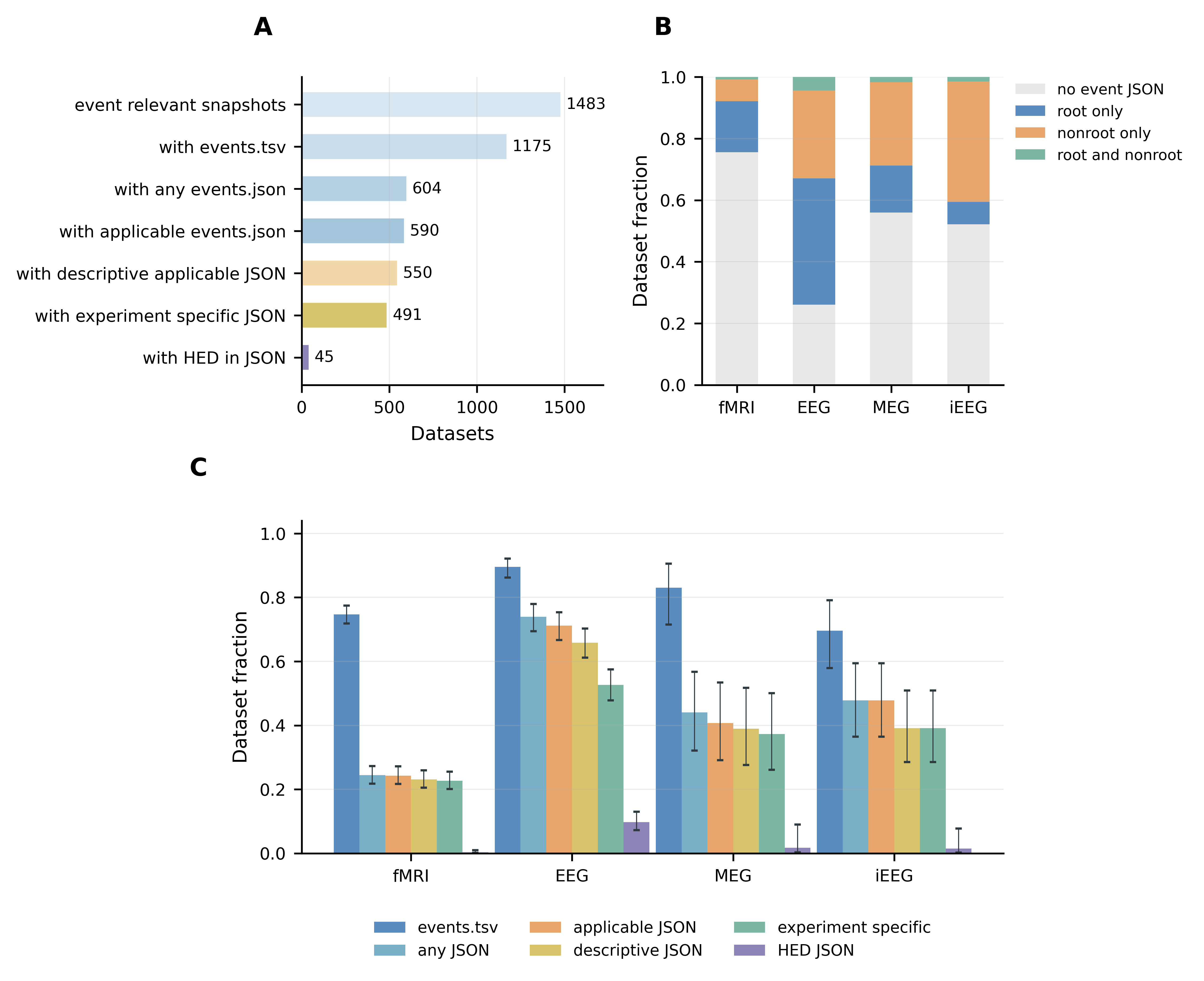

Supplement: Supplementary file 1 — Supplementary Material 1 Reproducibility package containing analysis scripts, requirements, derived CSV/JSON outputs, figures, manifest hashes, validation notes, and the manuscript source Markdown used to reproduce the reported audit tables and figures (ZIP 898 KB) [file 12021_2026_9797_MOESM1_ESM.zip › results/figures/figure1_event_readiness.png]

**A**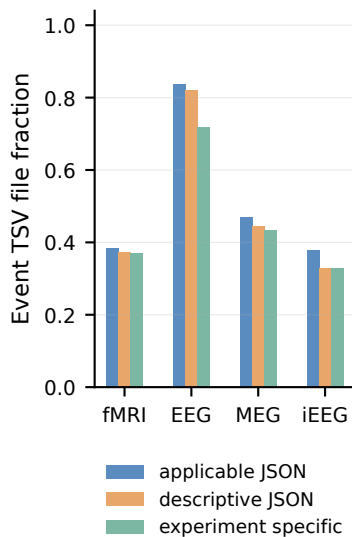**B**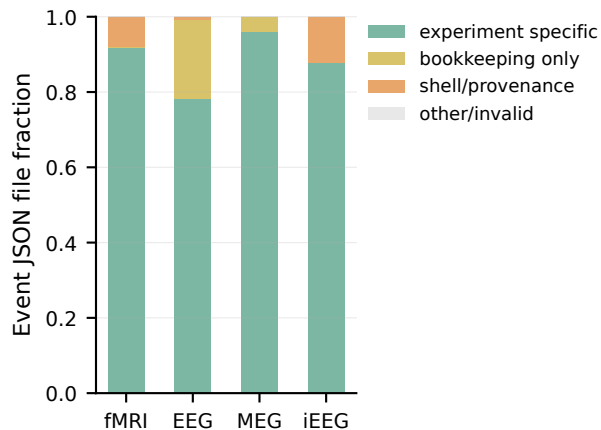**C**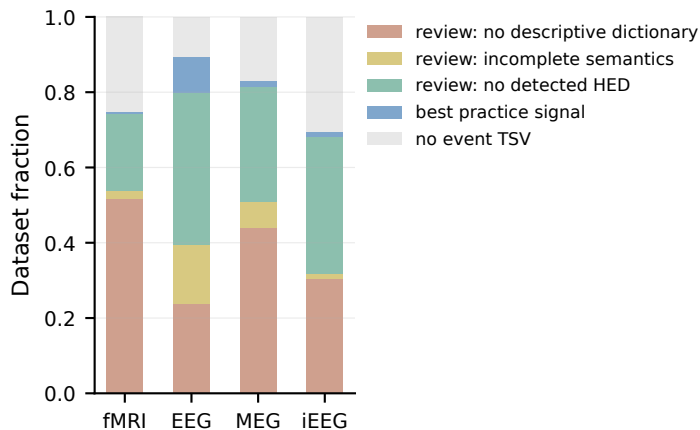**D**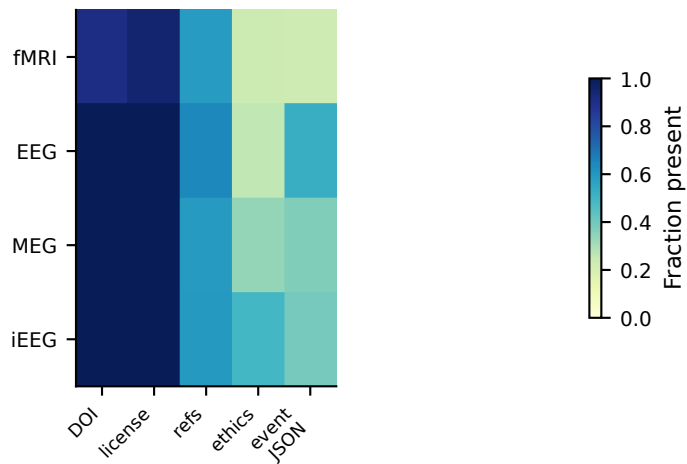

Supplement: Supplementary file 1 — Supplementary Material 1 Reproducibility package containing analysis scripts, requirements, derived CSV/JSON outputs, figures, manifest hashes, validation notes, and the manuscript source Markdown used to reproduce the reported audit tables and figures (ZIP 898 KB) [file 12021_2026_9797_MOESM1_ESM.zip › results/figures/figure2_metadata_validation.pdf]

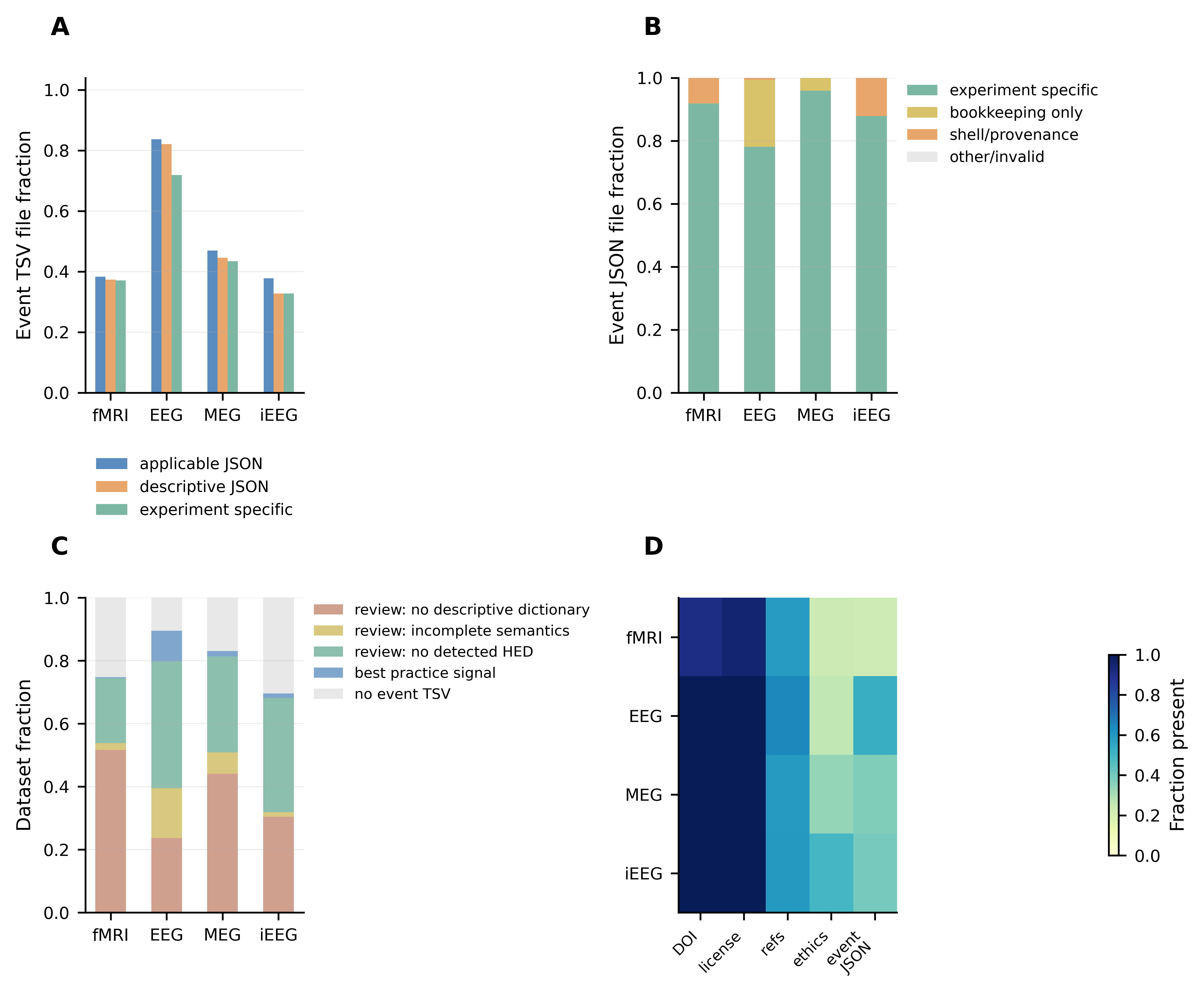

Supplement: Supplementary file 1 — Supplementary Material 1 Reproducibility package containing analysis scripts, requirements, derived CSV/JSON outputs, figures, manifest hashes, validation notes, and the manuscript source Markdown used to reproduce the reported audit tables and figures (ZIP 898 KB) [file 12021_2026_9797_MOESM1_ESM.zip › results/figures/figure2_metadata_validation.png]
